# Efficacy of Antimicrobial Treatment in Dogs with Atopic Dermatitis: An Observational Study

**DOI:** 10.3390/vetsci9080385

**Published:** 2022-07-27

**Authors:** Evi I. Sofou, Svetlina Aleksandrova, Elisa Badulescu, Manolis Chatzis, Manolis Saridomichelakis

**Affiliations:** Clinic of Medicine, Faculty of Veterinary Science, University of Thessaly, Trikalon Str. 224, GR-43132 Karditsa, Greece; sv.aleksandrova@yahoo.fr (S.A.); elisa.badulescu@artvetderm.ro (E.B.); mchatzis@vet.uth.gr (M.C.); msarido@vet.uth.gr (M.S.)

**Keywords:** allergy, antimicrobials, canine, dermatitis, efficacy, infection, *Malassezia*, pruritus, *Staphylococcus*

## Abstract

**Simple Summary:**

Atopic dermatitis (AD) is a very common allergic skin disease of dogs that is usually accompanied by skin infections, bacterial, fungal, or both. Although treatment of bacterial and fungal infections in dogs with AD has been recommended and is widely practiced, there are only a few studies evaluating its efficacy. The aim of this study was to evaluate the change in the severity of skin lesions and pruritus after the administration of systemic antimicrobials that resulted in the resolution of the infections. In total, 39 dogs were used, and treatment was prescribed according to the laboratory findings. For the evaluation of the skin lesions and pruritus, validated scales were used, and the scores before and after the treatment were compared. The severity of skin lesions and pruritus decreased significantly, by 30% and 35%, respectively. The efficacy of antimicrobial treatment was assessed as good to excellent by the clinician and the owner in 55% and 60% of the dogs, respectively. There was high variability in the response to treatment among dogs and further studies are needed to find factors that can predict the response to antimicrobial treatment in dogs with AD and skin infections.

**Abstract:**

There is a shortage of studies reporting the efficacy of antimicrobial treatment of dogs with atopic dermatitis (AD) and skin infections (SIs). The aim of this study was to evaluate the change in the severity of skin lesions and pruritus, and the overall efficacy of antimicrobial treatment, in dogs with AD and bacterial overgrowth/infection and/or *Malassezia* dermatitis. A total of 20 dogs with AD and SIs were prospectively enrolled (group A) and they were examined before and after the administration of systemic antimicrobials that resulted in the resolution of SIs. In addition, 19 dogs fulfilling the same inclusion criteria and treated with systemic, with or without topical antimicrobials, were included retrospectively (group B). Since there were no major differences between the groups, their results were combined. The severity of skin lesions decreased significantly, by 30% based on Canine Atopic Dermatitis Extent and Severity Index-4 (CADESI-4), by 28.1% based on the erythema domain of CADESI-4 and based on owner’s global assessment of the severity of skin lesions. Pruritus decreased significantly, by 34.7% based on the Pruritus Visual Analogue Scale (PVAS). The efficacy of antimicrobial treatment was assessed as good to excellent by the investigator and the owner in 55% and 60% of the dogs, respectively. Despite the significant improvement, there was high variability in the response to treatment among dogs. Further studies are needed to find factors that determine the response to antimicrobial treatment in dogs with AD and SIs.

## 1. Introduction

Canine atopic dermatitis (AD) is a chronic inflammatory skin disease of multifactorial pathogenesis, including a complex interplay among genetic predisposition, sensitization to environmental and/or food allergens, aberrant immune responses, skin barrier dysfunction, and microbial dysbiosis [1,2,3,4,5,6,7]. A healthy cutaneous microbiome is of critical importance in shaping the immunological responses of the host and for the protection from deleterious environmental insults [8]. A hallmark of human AD is the dramatic increase in the relative abundance of *Staphylococcus* spp. (mainly *S. aureus*) during flares, the positive correlation between the severity of skin lesions and the density of *Staphylococcus* spp. on the skin, and the development of impetigo lesions from whom *S. aureus* is usually cultured, whereas *Malassezia* spp. yeasts also play an important role in the development and aggravation of clinical signs [9]. Similarly, canine AD is characterized by bacterial and fungal dysbiosis that becomes more profound before flares and is partially restored during periods of remission [10,11,12,13,14,15]. This dysbiosis, along with the defective epidermal barrier and the chronic inflammation, is considered responsible for the frequent development of bacterial overgrowth, superficial and, less commonly, deep bacterial pyoderma, usually due to *Staphylococcus pseudintermedius*, as well as *Malassezia* dermatitis, in dogs with AD [2,11,16,17]. Furthermore, bacteria (most commonly *S. pseudintermedius*) and *Malassezia* yeasts stimulate flares of canine AD by increasing the production and release of pruritogenic and inflammatory cytokines and by producing microbial allergens [6], thus creating a vicious cycle.

Although treatment of bacterial overgrowth/infection and of *Malassezia* dermatitis in dogs with AD has been recommended [18,19,20] and is widely practiced, there are few studies evaluating its efficacy [11,21,22,23], and none of them report all currently proposed outcome measures for therapeutic trials on canine AD, including the improvement of skin lesions, the improvement of pruritus, and the owner’s assessment of treatment efficacy [24]. Thus, the aim of this open, observational, combined prospective, and retrospective study was to evaluate the changes in the severity of skin lesions and pruritus, as well as the investigator’s and owner’s assessment of the efficacy of antimicrobial treatment in dogs with AD and skin infections.

## 2. Materials and Methods

### 2.1. Prospective Study

A total of 20 client-owned dogs with non-seasonal AD, bacterial overgrowth/infection, and/or *Malassezia* dermatitis were prospectively enrolled (group A; Table S1). Inclusion criteria were: (a) a diagnosis of AD by a board-certified veterinary dermatologist (MS) based on history, clinical signs, exclusion of other pruritic skin diseases, and fulfillment of at least 6 out of the 8 set #1 diagnostic criteria proposed by Favrot et al. [25]; (b) clinical and cytological (impression smear and/or adhesive tape strip preparations) evidence of skin infections [26,27,28], severe and extensive enough to justify the administration of systemic antimicrobials (Table S2); (c) no pregnancy, lactation, or concurrent unrelated skin or systemic diseases; (d) no otitis severe enough to necessitate topical and/or systemic treatment; (e) no recent administration of systemic or topical medications that could have reduced the extent and severity of skin lesions and the severity of pruritus at enrolment. To this aim, dogs should not have been treated with oral H1 antihistamines during the previous 1 week and with topical glucocorticoids or oral short-acting glucocorticoids for the previous 2 weeks (i.e., for the optimal withdrawal period of these medications before intradermal testing) [29], with long-acting injectable glucocorticoids for the previous 8 weeks (i.e., for double of the minimum withdrawal period of these medications before intradermal testing) [29], with cyclosporine, topical tacrolimus, oclacitinib, pentoxifylline, ketoconazole or essential fatty acids for the previous 2 weeks, and with injectable lokivetmab for the previous 6 weeks; and (f) no current interventions to reduce exposure to possible allergens (e.g., house dust mite avoidance measures, elimination diet), and no current allergen immunotherapy.

To reduce, as much as possible, the reliance on owner compliance and dog cooperation with topical treatment, only systemic antimicrobials were prescribed. For dogs with an increased probability of bacterial infection caused by resistant organisms (i.e., administration of penicillins, cephalosporines, or fluoroquinolones during the previous 6 months, history of poor response to previous antibacterial courses, previous diagnosis of infection by resistant bacteria, cohabitation with other dogs with known infections caused by resistant bacteria, deep infection) systemic antibacterials were selected based on culture and susceptibility test, whereas the remaining dogs were treated empirically with either clindamycin (target dose: 11 mg/kg, once daily, per os) or amoxicillin–clavulanate (target dose: 20–25 mg/kg, twice daily, per os) (Table S2). The duration of the treatment was 1 week after resolution of bacterial overgrowth or superficial bacterial pyoderma and 2 weeks after resolution of deep bacterial pyoderma [30,31,32]. *Malassezia* dermatitis was treated with itraconazole (target dose: 5–10 mg/kg, once daily, per os) for 3–4 weeks [27]. No additional treatments, except prevention for ectoparasites, and no diet changes were allowed for the duration of the study. After the end of the antibacterial and/or antifungal course, the resolution of the infections was confirmed clinically and cytologically (Table S2).

On the day of enrollment (time 0), the following data were collected: (a) the extent and severity of skin lesions, assessed by the investigators using the validated Canine Atopic Dermatitis Extent and Severity Index-4 (CADESI-4) [33]; (b) the extent and severity of erythema (CADESI-4-E), one of the three domains of CADESI-4, was extracted from CADESI-4 recording forms, because changes in CADESI-4-E may be more representative of the efficacy of short-term (i.e., lasting <6 weeks) therapeutic interventions in dogs with AD compared to the changes of CADESI-4 [34]; c) the severity of skin lesions, assessed by the owner using the non-validated Owner’s Global Assessment of Severity (OGA-S) scale (1—absence of lesions, 2—mild lesions, 3—moderate lesions, or 4–extensive lesions) [33,35]; and d) the severity of pruritus, assessed by the owner using the validated Pruritus Visual Analogue Scale (PVAS) [36,37].

When the resolution of infections was confirmed (time 1), the following data were collected: (a) CADESI-4, assessed by the same investigator as at time 0; (b) CADESI-4-E, extracted from CADESI-4 recording forms as at time 0; (c) OGA-S, assessed by the same owner as at time 0; (d) PVAS, assessed by the same owner as at time 0; (e) the overall clinical improvement, assessed by one of the investigators using the non-validated Investigator’s Global Assessment of Efficacy (IGA-E) scale (0—no response, 1—poor response, 2—fair response, 3—good response, or 4—excellent response) [33]; and (f) the overall clinical improvement, assessed by the owner using the non-validated Owner’s Global Assessment of Treatment Efficacy (OGATE) scale (0—no response, 1—poor response, 2—fair response, 3—good response, or 4—excellent response) [24].

The following outcome measures were calculated: (a) the percentage (%) change of CADESI-4 between time 0 and time 1; (b) the % of dogs with CADESI-4 at time 0 > 10 (indicating mild (CADESI-4: 10–34), moderate (CADESI-4: 35–59) or severe (CADESI-4: ≥60) AD) and with CADESI-4 at time 1 in the range of normal dogs (CADESI-4-N: <10) [24,33]; (c) the % of dogs with CADESI-4 at time 0 > 34 (indicating moderate-to-severe AD) and with CADESI-4 at time 1 <35 (in the range of normal dogs or dogs with mild AD; CADESI-4-N2M) [24]; (d) the % change of CADESI-4-E between time 0 and time 1; (e) the change of OGA-S between time 0 and time 1; (f) the % change of PVAS between time 0 and time 1; (g) the % of dogs with PVAS at time 0 >1.9 (indicating mild (PVAS: 2–3.5), moderate (PVAS: 3.6–5.5) or severe (PVAS: ≥5.6) AD) and with PVAS at time 1 in the range of normal dogs (PVAS-N: <2) [24,37,38]; (h) the % of dogs with PVAS at time 0 >3.5 (indicating moderate-to-severe AD) and with PVAS at time 1 <3.6 (in the range of normal dogs or of dogs with mild AD; PVAS-N2M) [24]; (i) the % of dogs with IGA-E score of 3 (good response) or 4 (excellent response); and (j) the % of dogs with OGATE score of 3 (good response) or 4 (excellent response) [24].

### 2.2. Retrospective Study

Using a placebo-treated group of dogs with AD was considered unethical. Therefore, to partially account for the bias of the investigators and the owners who knew that they are participating in a clinical trial, the medical records of the clinic were searched for dogs with AD fulfilling the same inclusion criteria as group A. These dogs had been treated with systemic antibacterial and/or antifungal medications, following the same principles (selection of antimicrobials, treatment duration, no concurrent medications except ectoparasiticides, confirmation of the resolution of the infection after the end of the treatment) as in group A, with the only exception that concurrent topical antimicrobial treatment was permitted. A total of 19 clinical records fulfilling all the above criteria were found (group B; Tables S1 and S2).

Available data for group B dogs included CADESI-4, CADESI-4-E, and PVAS at time 0 and time 1. Subsequently, the same outcome measures, as in group A, were calculated except for the change of OGA-S between time 0 and time 1, and the % of dogs with IGA-E or OGATE scores of 3 or 4.

### 2.3. Statistical Methods

Statistical analyses were performed with IBM^®^ SPSS^®^ Statistics 23.0 for Windows and the level of significance was set at 5%. Categorical data were compared between group A and group B dogs using Pearson’s χ^2^ or Fischer’s exact test. The distribution of ordinal and continuous data was tested with Lilliefors’ modification of Kolmogorov–Smirnov test. Data following normal distribution are presented as mean ± standard deviation (SD), while data not following normal distribution are presented as median and range. For their comparison between group A and group B dogs, independent samples t-test or Mann–Whitney *U* test was used, depending on their distribution (normal or non-normal, respectively). For their comparison between time 0 and time 1, paired-samples t-test (normal distribution) or related-samples Wilcoxon signed-rank test (non-normal distribution) were used.

## 3. Results

The sex, breed, and age of group A and group B dogs are presented in Table S1. There were no significant differences in the distribution of sex (male or female; *p* = 0.855) or breed (purebred or mixed breed; *p* = 0.237), or age (*p* = 0.245) between the two groups, and their data were combined. Of the 39 dogs, 17 (43.6%) were male (7 neutered) and 22 (56.4%) were female (17 spayed). Twenty-nine dogs (74.4%) were purebred, and 10 (25.6%) dogs were mixed breed. Their age was 4.88 ± 2.71 years. 

The type of infection (bacterial overgrowth, superficial pyoderma, deep pyoderma, and/or *Malassezia* dermatitis), the method of selection of systemic antibacterials, and the number of days between time 0 and time 1 are presented in Table S2. There was no difference between the two groups in the % of dogs with bacterial infection, with *Malassezia* dermatitis, or both (*p* = 0.135), in the % of dogs with bacterial overgrowth, with superficial pyoderma, or with deep pyoderma (*p* = 1), in the % of dogs with a bacterial infection that was treated with empirically selected systemic antibacterials or with systemic antibacterials selected based on culture and susceptibility test (*p* = 0.648), or in the number of days between time 0 and time 1 (*p* = 0.365). Therefore, the data of the two groups were combined. Of the 39 dogs, 4 (10.3%) were diagnosed with bacterial overgrowth/infection, 11 (28.2%) with *Malassezia* dermatitis, and 24 (61.5%) with both. Of the 28 dogs with bacterial overgrowth/infection, 14 (50%) were diagnosed with bacterial overgrowth, 12 (42.9%) with superficial pyoderma, and 2 (7.1%) with deep pyoderma (when more than one type of bacterial overgrowth/infection was present in the same dog, the deepest one was recorded). For the treatment of these 28 dogs, systemic antibacterials were selected empirically (22/28—82.1%) or based on culture and susceptibility test (6/28–17.9%), and they included amoxicillin–clavulanate (19/28—67.9%; actual dose: 16.9–27.6 mg/kg, twice daily, per os), clindamycin (8/28–28.6%; actual dose: 10–12.3 mg/kg, once daily, per os) or enrofloxacin (1/28–3.6%; 10 mg/kg, once daily, per os). For the treatment of *Malassezia* dermatitis (35/39 dogs), itraconazole was prescribed at an actual dose of 5.6–10 mg/kg, once daily, per os. In addition, an antimicrobial shampoo (chlorhexidine 2%—miconazole 2%; Malaseb shampoo, Dehra, UK) was prescribed in 17/19 (89.5%) group B dogs, and the owners were instructed to use it 2–3 days/week. The median number of days between time 0 and time 1, and thus the duration of the treatment, was 29 (range: 21–47).

**Figure 1:**
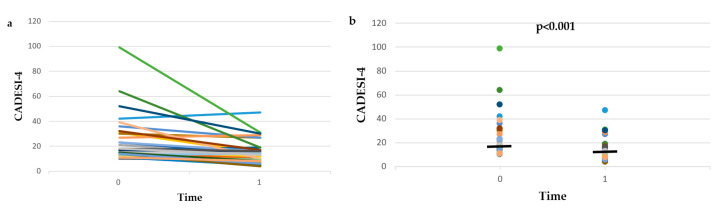
Line plot (**a**) and dot plot (**b**) of Canine Atopic Dermatitis Extent and Severity Index-4 (CADESI-4) scores before (time 0) and after (time 1) treatment of infections in the 20 dogs with atopic dermatitis that were included in the prospective study (group A) and the 19 dogs with atopic dermatitis that were included in the retrospective study (group B). The horizontal lines in the dot plot (**b**) represent median values.

The CADESI-4 scores of group A and group B dogs at time 0 and time 1, as well as the % change of CADESI-4 between time 0 and time 1 are presented in Figure 1 and Table S3. There was no difference between the two groups in the CADESI-4 scores at time 0 (*p* = 0.127) or time 1 (*p* = 0.127), or in the % change of CADESI-4 between time 0 and time 1 (*p* = 0.306). Therefore, the data of the two groups were combined.

CADESI-4 scores decreased significantly (*p* < 0.001), from 17 (range 10–99) at time 0 to 13 (range: 4–47) at time 1. The % reduction of CADESI-4 between time 0 and time 1 was 30 ± 24.8%. 

At time 0, 38/39 dogs had CADESI-4 scores >10 and of these dogs 10/38 (26.3%) had CADESI-4 scores <10 (i.e., CADESI-4-N) at time 1. The % of dogs with CADESI-4-N at time 1 was significantly higher (*p* = 0.027) in group B (8/18—44.4%) compared to group A (2/20—10%).

At time 0, 6/39 dogs had CADESI-4 scores >34 and of these dogs, 5/6 (83.3%) had CADESI-4 scores <35 (i.e., CADESI-4-N2M) at time 1. The % of dogs with CADESI-4-N2M at time 1 did not differ (*p* = 0.5) between group A (2/3—66.7%) and group B (3/3—100%).

**Figure 2:**
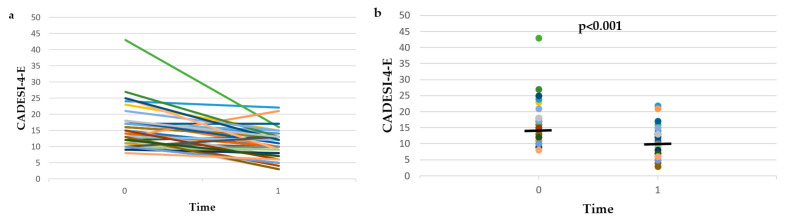
Line plot (**a**) and dot plot (**b**) of the erythema domain of Canine Atopic Dermatitis Extent and Severity Index-4 (CADESI-4-E) scores before (time 0) and after (time 1) treatment of infections in the 20 dogs with atopic dermatitis that were included in the prospective study (group A) and the 19 dogs with atopic dermatitis that were included in the retrospective study (group B). The horizontal lines in the dot plot (**b**) represent median values.

The erythema domain of CADESI-4 (i.e., CADESI-4-E) scores of group A and group B dogs at time 0 and time 1, as well as the % change of CADESI-4-E between time 0 and time 1 are presented on Figure 2 and Table S4. Although CADESI-4-E was significantly higher in group A compared to group B at both time 0 (*p* = 0.019) and time 1 (*p* = 0.003), the % change of CADESI-4-E between time 0 and time 1 did not differ between groups (*p* = 0.257). Therefore, the data of the two groups were combined. CADESI-4-E scores decreased significantly (*p* < 0.001) between time 0 and time 1, and the % reduction of CADESI-4-E between time 0 and time 1 was 28.1 ± 28.6%.

The severity of skin lesions assessed by the owner (i.e., OGA-S) of group A dogs at time 0 and time 1, as well as the numerical change of OGA-S between time 0 and time 1 are presented in Table S5. At time 0 median OGA-S was 3 (range: 2–4), at time 1 it was 2 (range: 1–4) and the difference was significant (*p* = 0.003).

**Figure 3:**
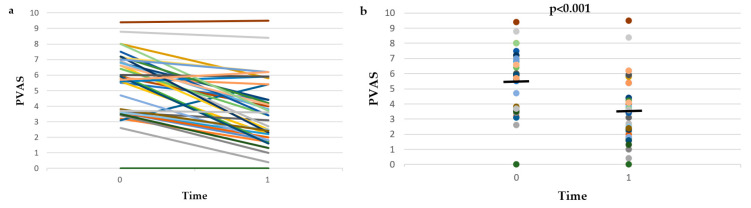
Line plot (**a**) and dot plot (**b**) of Pruritus Visual Analogue Scale (PVAS) scores before (time 0) and after (time 1) treatment of infections in the 20 dogs with atopic dermatitis that were included in the prospective study (group A) and the 19 dogs with atopic dermatitis that were included in the retrospective study (group B). The horizontal lines in the dot plot (**b**) represent mean values.

The PVAS scores of group A and group B dogs at time 0 and time 1, as well as the % change of PVAS between time 0 and time 1 are presented in Figure 3 and Table S6. PVAS scores at time 1 were significantly higher in group B compared to group A dogs (*p* = 0.025), but there was no difference between groups in the PVAS scores at time 0 (*p* = 0.076) or in the % change of PVAS between time 0 and time 1 (*p* = 0.435). Therefore, the data of the two groups were combined.

PVAS scores decreased significantly (*p* < 0.001), from 5.5 ± 2 at time 0 to 3.6 ± 2.1 at time 1. The % reduction of PVAS between time 0 and time 1 was 34.7 ± 31.5%. 

At time 0, 38/39 dogs had PVAS scores >1.9, and of these dogs 9/38 (23.7%) had PVAS scores <2 (i.e., PVAS-N) at time 1. The % of dogs with PVAS-N at time 1 did not differ (*p* = 1) between group A (5/19—26.3%) and group B (4/19—21.1%).

At time 0, 33/39 dogs had PVAS scores >3.5 and of these dogs 16/33 (48.5%) had PVAS scores <3.6 (i.e., PVAS-N2M) at time 1. The % of dogs with PVAS-N2M at time 1 was significantly higher (*p* = 0.024) in group A (11/16—38.8%) compared to group B (5/17—29.4%).

The assessment of the overall efficacy of antimicrobial treatment by the investigator (i.e., IGA-E) and the owner (i.e., OGATE) of group A dogs is presented in Table S7. An IGA-E score of 3 or 4, indicative of a good or excellent response to treatment, was given to 11/20 (55%) dogs and an OGATE score of 3 or 4 was given to 12/20 (60%) dogs.

## 4. Discussion

The results of this combined prospective and retrospective observational study show that after the treatment of skin infections, the lesional scores of dogs with AD were significantly reduced, by 30 ± 24.8% based on CADESI-4, by 28.1 ± 28.6% based on CADESI-4-E, and by one grade based on OGA-S. At the same time, in 26.3% of the dogs, CADESI-4 scores decreased from the range of dogs with AD into the range of normal dogs, and in 83.3% of them, CADESI-4 scores decreased from the range of dogs with moderate-to-severe AD into the range of normal dogs or dogs with mild AD. In addition, PVAS scores decreased significantly (34.7 ± 31.5% reduction), in 23.7% of the dogs PVAS scores decreased from the range of dogs with AD into the range of normal dogs, and in 48.5% of them, PVAS scores decreased from the range of dogs with moderate-to-severe AD into the range of normal dogs or dogs with mild AD. Finally, the response to antimicrobial treatment was graded as good or excellent by the investigators and the owners of 55% and 60% of the dogs, respectively. These results show that, on a population basis, dogs with AD and skin infections (bacterial overgrowth/infection and/or *Malassezia* dermatitis) benefit from antimicrobial treatment. However, the high SDs of the % changes of CADESI-4, CADESI-4-E, and PVAS imply a high interindividual variability in the response to antimicrobial treatment, which is further exemplified by the visual inspection of Figure 1, Figure 2 and Figure 3 and Tables S3–S7. Indeed, despite the average improvement, CADESI-4 scores remained unchanged or increased after treatment in 17.9% of the dogs (Table S3), CADESI-4-E scores remained unchanged or increased in 12.8% of the dogs (Table S4), 30% of the owners considered that skin lesions remained stable or became worse (Table S5), PVAS scores remained unchanged or increased after treatment in 12.8% of the dogs (Table S6), and in 25% and 30% of the dogs the response to treatment was graded as “no” or “poor” by the investigators and the owners, respectively. 

The diagnosis of AD sensu lato and skin infections was based on the currently accepted criteria [26,27,39] and all dogs had to fulfill at least 6 out of the 8 set #1 diagnostic criteria of Favrot et al. [25], as it has been proposed for the enrollment of dogs with AD in clinical trials [39,40]. However, the latter criteria apply to dogs with the “typical” phenotype of AD and they tend to exclude dogs with “atypical” AD [40,41]. Therefore, it is unknown if the results of the present study also apply to dogs with AD not presenting the typical clinical features of the disease. In addition, proof of sensitization to environmental and/or food allergens was not an inclusion criterion. Subsequently, our results are only applicable to dogs with AD sensu lato, and we cannot exclude the possibility that dogs with AD and environmental allergy, dogs with AD and food allergy, or dogs with idiopathic AD [1] may respond differently to antimicrobial treatment. 

The optimal withdrawal period of the medications that can lower the severity of skin lesions and pruritus, before the enrolment of dogs with AD into clinical trials, has not been determined. We decided to adopt the optimal withdrawal periods and to double the minimum withdrawal periods before the intradermal test, when applicable [29], and for the remaining anti-inflammatory and antipruritic medications we selected a 2-week withdrawal period (same as for topical and oral-short acting glucocorticoids), with the exception of lokivetmab (6 weeks) due to its long duration of action [42]. Although these time periods are similar to those in most previous therapeutic trials in dogs with AD, we cannot exclude the possibility of a carry-over effect of previous medications, which would have a more pronounced effect on time 0 scores compared to time 1 scores. In this case, our results may have underestimated the efficacy of antibacterial treatment. On the other hand, the anti-inflammatory properties of itraconazole [43], which was administered to 35/39 dogs, and enrofloxacin [44], which was administered to 1/39 dogs, may have been partially responsible for the recorded efficacy. Finally, it is widely accepted that open trials tend to overestimate the efficacy of therapeutic interventions in canine AD. The lack of major differences in the outcome measures between prospectively enrolled (group A) and retrospectively used (group B) dogs, may imply that knowledge of participation in a clinical trial did not induce a systematic bias in the investigators and the owners of group A dogs, but does not negate the possible influence of other factors, such as the remissions of canine AD that occur irrespectively of therapeutic interventions. Taking into consideration all these parameters, the authors believe that the actual benefit of dogs with AD from the treatment of their infections is probably similar or somehow lower than the one found in the present study. 

Currently, there is no single outcome measure able to fully characterize the efficacy of therapeutic interventions in dogs with AD, because each outcome measure has its own advantages and limitations. For this reason, a set of outcome measures, such as CADESI-4-N or CADESI-4-N2M, PVAS-N or PVAS-N2M, and OGATE, has been recommended for clinical trials (typically randomized and controlled) lasting ≥6 weeks [24]. In the absence of a similar set for open trials of shorter duration, we selected to use, in addition to all the above, the % change of CADESI-4, CADESI-4-E, and PVAS (with the understanding that the clinical importance of these changes depends on CADESI-4, CADESI-4-E, and PVAS scores at time 0, respectively), as well as the OGA-S and the IGA-E that, despite being non-validated scales, have been used for the validation of CADESI-4 [33]. Essentially, we report our results in terms of all available outcome measures and in a way that will enable their use in future meta-analyses, when and if they will be performed.

Despite the large number of variables, related to signalment, type of infection, treatment, and severity of AD at time 0, that have been compared between group A and group B dogs, only one difference was found: CADESI-4-E was significantly higher in group A. This difference was likely due to chance, and it did not affect the results of the study, because CADESI-4-E was also significantly higher in group A dogs at time 1 and, most importantly, because the related outcome measure (i.e., % change of CADESI-4-E between time 0 and time 1) did not differ between the groups.

Two out of the seven outcome measures that were applicable to both groups of dogs differ between them: the % of dogs with CADESI-4-N was significantly higher in group B and the % of dogs with PVAS-N2M was significantly higher in group A (in parallel with the significantly lower PVAS scores of group A dogs at time 1). Perhaps some of the nine dogs in group B that were diagnosed only with bacterial overgrowth/infection or only with *Malassezia* dermatitis (Table S2) had a concurrent undiagnosed yeast or bacterial infection, respectively, which was effectively treated with the chlorhexidine-miconazole shampoo and/or the shampoo may have a beneficial effect on microbial dysbiosis [13], thus explaining the higher % of dogs with CADESI-4-N in group B. On the other hand, this shampoo may be irritant and can cause increased transepidermal water loss [23] that can lead to xerosis and increased pruritus, thus explaining the lower % of dogs with PVAS-N2M (and the higher PVAS scores at time 1) in group B.

In a randomized controlled trial investigating the efficacy of an oral fatty acid, vitamin and zinc supplement in 26 dogs with AD sensu lato, which were already controlled with prednisolone or cyclosporine, the administration of systemic antimicrobials for the treatment of bacterial and yeast infections that occurred during the trial was not associated with a significant improvement of skin lesions (assessed by Canine Atopic Dermatitis Lesion Index) or with a clear-cut reduction of PVAS [22]. In an open trial, the administration of systemic antibacterials in 14 dogs with AD sensu lato and superficial bacterial pyoderma for 4–6 weeks did not result in the significant improvement of skin lesions that were assessed by a modified regional CADESI-3 (i.e., the older iteration of CADESI that has different sensitivity to change compared to CADESI-4) scoring system [11]. In another open trial, weekly bathing with chlorhexidine ± miconazole shampoo of six dogs with AD sensu lato, history of superficial bacterial pyoderma, and current colonization by methicillin-resistant *Staphylococcus* spp. resulted in non-significant changes in CADESI-3 but in a significant reduction of pruritus after 4 weeks [21]. Finally, in a randomized controlled trial comparing two topical treatments in 16 dogs with AD sensu lato and *Malassezia* dermatitis, one out of the two treatments resulted in a significant reduction of CADESI-4, and both resulted in a significant reduction of PVAS after 4 weeks [23]. Differences in the design, the concurrent medications, and (with the exception of the latter study) the reported outcome measures complicate the comparison of our results to those of these previous studies.

Intentionally, we did not perform statistical analyses to search for possible correlations between the response to antimicrobial treatment and factors such as the signalment of the dogs, the severity of AD at time 0, the type (bacterial, yeast, or both), and the depth of the infection. This was decided due to the open design of our study, the low number of dogs with only bacterial infection (4/39), and the lack of data on multiple variables that may determine the efficacy of antimicrobial treatment, such as the extent of cutaneous dysbiosis, the predominant bacterial and yeast species and strains at time 0, their virulence factors, their relative susceptibility to antimicrobials, and the immunological responses of the dogs to these organisms [10,11,13,15,45,46]. Further controlled studies are needed to investigate these factors and to find markers able to detect dogs with AD and skin infections that are not expected to benefit from antimicrobial treatment. Furthermore, for the dogs with AD that benefit from this type of treatment, the efficacy of systemic antimicrobials, topical antiseptics/antifungals, alternative topical treatments (such as natural oils) [23,45], systemic and topical anti-inflammatory drugs, and their combinations must be compared, keeping in mind that systemic antibacterials result in only a temporal normalization of dysbiosis [11] and promote bacterial resistance [47], and that topical antiseptics/antifungals may deteriorate the already compromised epidermal barrier [23]. Finally, the duration of systemic antimicrobial treatment may have to be reconsidered [48]. 

## 5. Conclusions

Administration of systemic antibacterials and/or itraconazole with or without topical treatment with chlorhexidine 2%—miconazole 2% shampoo resulted in a significant reduction of skin lesions and pruritus in 39 dogs with AD and bacterial and/or *Malassezia* dermatitis. After treatment, 26.3% (10/38) of the dogs had skin lesional scores compatible with normal dogs and 83.3% (5/6) compatible with normal dogs or dogs with mild AD; 23.7% (9/38) of the dogs had pruritus scores compatible with normal dogs and 48.5% (16/33) compatible with normal dogs or dogs with mild AD. In addition, the efficacy of the treatment was scored as good or excellent by the investigators and the owners of 55% and 60% of the dogs, respectively. However, the efficacy of the treatment varied among dogs and a substantial proportion of them did not have a clear benefit or even deteriorated during treatment.

## Data Availability

The data analyzed for the study are available from the corresponding author upon reasonable request.

## Supplementary Materials

The following supporting information can be downloaded at: https://www.mdpi.com/article/10.3390/vetsci9080385/s1, Table S1: Sex, breed, and age (in years) of the 20 dogs with atopic dermatitis that were included in the prospective study (group A) and of the 19 dogs with atopic dermatitis that were included in the retrospective study (group B); Table S2: Type of infection (bacterial overgrowth, superficial pyoderma, deep pyoderma, and/or *Malassezia* dermatitis), selection of systemic antibacterials (empirical or based on culture and susceptibility test) and number of days between initial examination and re-examination (days (time 0—time1)) for the 20 dogs with atopic dermatitis that were included in the prospective study (group A) and the 19 dogs with atopic dermatitis that were included in the retrospective study (group B); Table S3: Canine Atopic Dermatitis Extent and Severity Index-4 (CADESI-4) scores before (time 0) and after (time 1) treatment of infections and percent change of CADESI-4 between time 0 and time 1 (% change CADESI-4) in the 20 dogs with atopic dermatitis that were included in the prospective study (group A) and the 19 dogs with atopic dermatitis that were included in the retrospective study (group B); Table S4: Erythema domain of Canine Atopic Dermatitis Extent and Severity Index-4 (CADESI-4-E) scores before (time 0) and after (time 1) treatment of infections and percent change of CADESI-4-E between time 0 and time 1 (% change CADESI-4-E) in the 20 dogs with atopic dermatitis that were included in the prospective study (group A) and the 19 dogs with atopic dermatitis that were included in the retrospective study (group B); Table S5: Owner’s Global Assessment of Severity (OGA-S) of skin lesions scores before (time 0) and after (time 1) treatment of infections and numerical change of OGA-S between time 0 and time 1 in the 20 dogs with atopic dermatitis that were included in the prospective study (group A); Table S6: Pruritus Visual Analogue Scale (PVAS) scores before (time 0) and after (time 1) treatment of infections and percent change of PVAS between time 0 and time 1 (% change PVAS) in the 20 dogs with atopic dermatitis that were included in the prospective study (group A) and the 19 dogs with atopic dermatitis that were included in the retrospective study (group B); Table S7: Investigator’s Global Assessment of Efficacy (IGA-E) and Owner’s Global Assessment of Treatment Efficacy (OGATE) after treatment of infections in the 20 dogs with atopic dermatitis that were included in the prospective study (group A).
