# Supplementary material for: Efficacy of Antimicrobial Treatment in Dogs with Atopic Dermatitis: An Observational Study
Source: Vet Sci. 2022 Jul 27;9(8):385. doi: 10.3390/vetsci9080385 (PMC9332798; doi:10.3390/vetsci9080385)
Supplement: Supplementary file 1 [file vetsci-09-00385-s001.zip › Table S1.pdf]

veterinary sciences

MDPI

Table S1: sex, breed, and age (in years) of the 20 dogs with atopic dermatitis that were included in the prospective study (group A) and of the 19 dogs with atopic dermatitis that were included in the retrospective study (group B).

Dog #	Sex	Breed	Age
		Prospective study (group A)	
1	M	Bull terrier	10
2	MC	Mixed-breed	4
3	M	Poodle	5
4	M	Labrador retriever	4
5	F	Dogo Argentino	6
6	FS	Cavalier King Charles Spaniel	4
7	FS	Golden retriever	6
8	F	Mixed-breed	5
9	F	Labrador retriever	7.5
10	FS	Mixed-breed	6
11	FS	West Highland white terrier	5
12	FS	Pitbull terrier	4
13	MC	German shepherd	10
14	FS	Mixed-breed	3.5
15	MC	Mixed-breed	3.5
16	MC	French Bulldog	8
17	FS	Maltese terrier	6
18	M	Mixed-breed	4.5
19	MC	Labrador retriever	3
20	FS	Mixed-breed	2.5
		Retrospective study (group B)	
1	M	French Bulldog	2
2	FS	Poodle	8
3	FS	West Highland white terrier	3.5
4	M	German shepherd	9
5	M	Mixed-breed	1.1
6	F	Pekingese	4.5
7	M	Yorkshire terrier	1
8	FS	Staffordshire terrier	1
9	F	German shepherd	4
10	FS	Maltese terrier	7
11	M	Labrador retriever	3
12	FS	Yorkshire terrier	3
13	M	French Bulldog	3
14	MC	Mixed-breed	1.1
15	FS	Shih-tzu	13
16	FS	Shih-tzu	6
17	FS	Yorkshire terrier	3.5
18	MC	Greek hound	2.5
19	FS	Mixed-breed	6.5

* Abbreviations: F: female; FS: female-spayed; M: male; MC: male castrated.
