# Supplementary material for: Efficacy of Antimicrobial Treatment in Dogs with Atopic Dermatitis: An Observational Study
Source: Vet Sci. 2022 Jul 27;9(8):385. doi: 10.3390/vetsci9080385 (PMC9332798; doi:10.3390/vetsci9080385)
Supplement: Supplementary file 1 [file vetsci-09-00385-s001.zip › Table S2.pdf]

veterinary sciences

MDPI

Table S2: type of infection (bacterial overgrowth, superficial pyoderma, deep pyoderma and/or *Malassezia* dermatitis), selection of systemic antibacterials (empirical or based on culture and susceptibility test) and number of days between initial examination and re-examination [days (time 0-time1)] for the 20 dogs with atopic dermatitis that were included in the prospective study (group A) and the 19 dogs with atopic dermatitis that were included in the retrospective study (group B).

Dog #	Type of infection	Selection of antibacterial	Days (time 0-time 1)
	Prospective study (group A)		
1	SP + MD	C-S	47
2	MD		26
3	MD		34
4	SP + MD	E	32
5	MD		39
6	SP + MD	C-S	35
7	BO + MD	E	34
8	MD		30
9	MD		30
10	SP + MD	E	28
11	BO + MD	E	39
12	SP + MD	E	27
13	DP + MD	C-S	28
14	SP + MD	C-S	28
15	BO + MD	E	28
16	BO + MD	E	38
17	BO + MD	E	28
18	MD		28
19	BO + MD	E	29
20	BO + MD	E	36
	Retrospective study (group B)		
1	BO + MD	E	28
2	MD		28
3	MD		35
4	BO + MD	C-S	35
5	MD		42
6	DP	C-S	26
7	BO + MD	E	27
8	SP + MD	E	28
9	BO	E	27
10	BO + MD	E	35
11	SP	E	28
12	SP + MD	E	35
13	MD		27
14	SP	E	28
15	BO + MD	E	42
16	BO + MD	E	27
17	MD		35
18	SP + MD	E	36
19	SP + MD	E	21

* Abbreviations: BO: bacterial overgrowth; DP: deep pyoderma; MD: *Malassezia* dermatitis; SP: superficial pyoderma.
