# Supplementary material for: Efficacy of Antimicrobial Treatment in Dogs with Atopic Dermatitis: An Observational Study
Source: Vet Sci. 2022 Jul 27;9(8):385. doi: 10.3390/vetsci9080385 (PMC9332798; doi:10.3390/vetsci9080385)
Supplement: Supplementary file 1 [file vetsci-09-00385-s001.zip › Table S3.pdf]

veterinary sciences

MDPI

Table S3: Canine Atopic Dermatitis Extent and Severity Index-4 (CADESI-4) scores before (time 0) and after (time 1) treatment of infections and percent change of CADESI-4 between time 0 and time 1 (% change CADESI-4) in the 20 dogs with atopic dermatitis that were included in the prospective study (group A) and the 19 dogs with atopic dermatitis that were included in the retrospective study (group B).

Dog #	CADESI-4 (time 0)	CADESI-4 (time 1)	% change CADESI-4
Prospective study (group A)			
1	17	14	-17.6
2	18	10	-44.4
3	21	13	-38.1
4	31	15	-51.6
5	42	47	11.9
6	99	31	-68.7
7	16	13	-18.8
8	13	4	-69.2
9	11	10	-9.1
10	30	27	-10
11	17	19	11.8
12	17	17	0
13	36	27	-25
14	27	29	7.4
15	16	7	-56.3
16	17	12	-29.4
17	17	15	-11.8
18	14	10	-28.6
19	23	14	-39.1
20	15	10	-33.3
Median (range) or mean ± SD	17 (11-99)	14 (4-47)	-26 ± 24.6
Retrospective study (group B)			
1	10	10	0
2	11	7	-36.4
3	11	5	-54.6
4	64	19	-70.3
5	23	16	-30.4
6	39	10	-74.4
7	15	15	0
8	12	9	-25
9	13	14	7.7
10	13	9	-30.8
11	17	16	-5.9
12	32	17	-46.9
13	18	16	-11.1
14	14	4	-71.4
15	52	30	-42.3
16	15	8	-46.7
17	14	6	-57.1
18	11	8	-27.3
19	18	13	-27.8
Median (range) or mean ± SD	15 (10-64)	12.2 ± 6.2	-34.2 ± 25
Both groups			
Median (range) or mean ± SD	17 (10-99)	13 (4-47)	-30 ± 24.8

* Abbreviations: SD: standard deviation
