# Supplementary material for: Efficacy of Antimicrobial Treatment in Dogs with Atopic Dermatitis: An Observational Study
Source: Vet Sci. 2022 Jul 27;9(8):385. doi: 10.3390/vetsci9080385 (PMC9332798; doi:10.3390/vetsci9080385)
Supplement: Supplementary file 1 [file vetsci-09-00385-s001.zip › Table S5.pdf]

veterinary sciences

MDPI

Table S5: Owner's Global Assessment of Severity (OGA-S) of skin lesions scores * before (time 0) and after (time 1) treatment of infections and numerical change of OGA-S between time 0 and time 1 in the 20 dogs with atopic dermatitis that were included in the prospective study (group A).

**Dog #**	**OGA-S (time 0)**	**OGA-S (time 1)**	**Change of OGA-S**
1	2	2	0
2	2	2	0
3	4	1	-3
4	3	2	-1
5	3	2	-1
6	4	1	-3
7	3	2	-1
8	3	2	-1
9	2	1	-1
10	4	3	-1
11	3	2	-1
12	2	2	0
13	3	2	-1
14	2	3	1
15	3	1	-2
16	3	2	-1
17	3	1	-2
18	2	3	1
19	4	4	0
20	2	1	-1

* OGA-S scale: 1-absence of lesions, 2-mild lesions, 3-moderate lesions, 4-extensive lesions
