# Supplementary material for: Efficacy of Antimicrobial Treatment in Dogs with Atopic Dermatitis: An Observational Study
Source: Vet Sci. 2022 Jul 27;9(8):385. doi: 10.3390/vetsci9080385 (PMC9332798; doi:10.3390/vetsci9080385)
Supplement: Supplementary file 1 [file vetsci-09-00385-s001.zip › Table S7.pdf]

veterinary sciences

MDPI

Table S7: Investigator's Global Assessment of Efficacy (IGA-E) * and Owner's Global Assessment of Treatment Efficacy (OGATE) ** after treatment of infections in the 20 dogs with atopic dermatitis that were included in the prospective study (group A).

**Dog #**	**IGA-E**	**OGATE**
1	1	1
2	4	3
3	3	3
4	3	3
5	2	0
6	4	4
7	3	3
8	3	4
9	1	0
10	3	2
11	0	1
12	2	2
13	3	3
14	1	1
15	3	3
16	3	3
17	1	3
18	2	3
19	2	1
20	3	4

* IGA-E: 0-no response, 1-poor response, 2-fair response, 3-good response, 4-excellent response

** OGATE: 0-no response, 1-poor response, 2-fair response, 3-good response, or 4-excellent response
